# Outer retinal tubulation formation and clinical course of advanced age-related macular degeneration

**DOI:** 10.1038/s41598-021-94310-5

**Published:** 2021-07-19

**Authors:** Alessandro Arrigo, Emanuela Aragona, Ottavia Battaglia, Andrea Saladino, Alessia Amato, Federico Borghesan, Adelaide Pina, Francesca Calcagno, Rashid Hassan Farah, Francesco Bandello, Maurizio Battaglia Parodi

**Affiliations:** Department of Ophthalmology, IRCCS San Raffaele Scientific Insititute, via Olgettina 60, 20132 Milan, Italy

**Keywords:** Visual system, Biomarkers, Outcomes research

## Abstract

Outer retinal tubulations (ORT) are a relatively new finding characterizing outer retinal atrophy. The main aim of the present study was to describe ORT development in advanced age-related macular degeneration (AMD) and to assess its relationship with disease’s severity. Patients with advanced AMD characterized either by macular neovascularization or geographic atrophy, showing signs of outer retinal disruption or retinal pigment epithelium atrophy on structural optical coherence tomography (OCT) at the inclusion examination were prospectively recruited. All the patients underwent complete ophthalmologic evaluation, structural OCT scans and fundus autofluorescence imaging. The planned follow-up was of 3-years. Main outcome measures were ORT prevalence, mechanism of ORT formation, mean time needed for complete ORT formation, best-corrected visual acuity (BCVA), definitely decreased autofluorescence (DDAF) area, questionably decreased autofluorescence (QDAF) area, retinal layer thickness, foveal sparing, number of intravitreal injections. We also assessed the possible role of external limiting membrane (ELM) and Müller cells in ORT pathogenesis. Seventy eyes (70 patients) were included; 43 showed dry AMD evolving to geographic atrophy, while 27 displayed the features of wet AMD. Baseline BCVA was 0.5 ± 0.5 LogMAR, decreasing to 0.9 ± 0.5 LogMAR at the 3-year follow-up (*p* < 0.01). We detected completely formed ORT in 26/70 eyes (37%), subdivided as follows: 20 eyes (77%) wet AMD and 6 eyes (23%) dry AMD (*p* < 0.01). ORT took 18 ± 8 months (range 3–35 months) to develop fully. We described the steps leading to ORT development, characterized by progressive involvement of, and damage to the photoreceptors, the ELM and the RPE. Eyes displaying ORT were associated with a smaller QDAF area, less retinal layers damage and lower rate of foveal sparing than eyes free of ORT (*p* < 0.01). We also described pigment accumulations simulating ORT, which were detected in 16/70 eyes (23%), associated with a greater loss of foveal sparing, increased DDAF area and smaller QDAF area at the 3-year follow-up (*p* < 0.01). In conclusion, this study provided a description of the steps leading to ORT development in AMD. ELM and Müller cells showed a role in ORT pathogenesis. Furthermore, we described a subtype of pigment hypertrophy mimicking ORT, evaluating its clinical utility.

## Introduction

Outer retinal tubulations (ORT) represent a relatively recent structural optical coherence tomography (OCT) finding and was first described by Zweifel and colleagues in 2009^1^. The precise pathogenesis of ORT is still unknown, although previous studies suggested damaged photoreceptors and retinal pigment epithelium (RPE) cells played a primary role in ORT formation^1–3^. ORT has been previously described in several retinal diseases, including age-related macular degeneration (AMD), diabetic retinopathy, central serous chorioretinopathy and retinal dystrophies^4–7^. Although found in diseases characterized by completely different pathogeneses, there is an overall agreement in associating ORT with outer retinal degeneration and atrophy. The role of ORT in AMD is not yet fully understood; however, the presence of ORT is associated with AMD phenotypes characterized by different morpho-functional impairment, compared with AMD without ORT. Recently, the presence of ORT in GA due to non-neovascular AMD was associated with smaller impairment of the retinal vascular network^8^. Furthermore, a recent study by Janse van Rensburg and colleagues (2021) reported a stronger association between ORT onset and type 2 MNV, compared with the other MNV forms^9^. The aim of the study was to describe the development and the evolution of ORT in AMD, focusing on the changes in retinal structure detectable on structural OCT. The secondary goal was the assessment of the relationship between ORT and the further degenerative evolution of advanced AMD stages.

## Materials and methods

The study was designed as a prospective, observational case series, with a follow-up of a minimum of 3 years. We collected data from patients affected by advanced AMD recruited at the Ophthalmology Unit of IRCCS Scientific Institute San Raffaele Hospital, Milan, Italy. The patients provided signed, informed consent and the study was approved by the ethical committee of IRCCS Scientific Institute San Raffaele Hospital, in accordance with the Declaration of Helsinki.

The inclusion criteria were eyes affected by advanced AMD, characterized by either macular neovascularization (MNV) or geographic atrophy. Exclusion criteria included high media opacity, ophthalmologic surgery occurred six months before the inclusion into the study, history of nutraceutic supplementation longer than 6 months, and any other ophthalmologic or systemic condition (such as uncontrolled systemic arterial hypertensions, diabetes mellitus, glaucoma, uveitis, etc.) potentially affecting the analyses.

All the patients underwent complete periodic ophthalmological examinations, including best-corrected visual acuity (BCVA), using ETDRS charts, anterior and posterior slit-lamp examination, tonometry, structural OCT scans (HRA2 + OCT Spectralis Heidelberg, Heidelberg Engineering, Germany), and fundus autofluorescence. Eyes complicated by MNV were treated by anti-VEGF intravitreal injections, administered in accordance with a pro-re-nata regimen. The diagnosis of MNV was confirmed at the onset by means of fluorescein angiography and indocyanine green angiography.

Structural OCT scans included radial, raster and dense acquisitions with ART > 25 and enhanced depth imaging.

The quantitative structural OCT analysis, performed using the tool provided by Heidelberg software (Software Version 6.12.1, Heidelberg Engineering, Germany), involved measuring central macular thickness (CMT), retinal nerve fiber layer (RNFL) thickness, ganglion cell layer (GCL) thickness, inner plexiform layer (IPL) thickness, inner nuclear layer (INL) thickness, outer plexiform layer (OPL) thickness, outer nuclear layer (ONL) thickness, RPE thickness, choroidal thickness (CT), Sattler layer thickness (SLT), and Haller layer thickness (HLT). Two expert examiners (AA, EA) considered the following four points for each retinal layer: 750 µm (right-left) and 1500 µm (right-left). The mean value was considered as the final thickness. In the case of choroidal layers, we added a fifth measurement, performed in the subfoveal region.

The same experts (AA, EA) measured the area of definitely decreased autofluorescence (DDAF) and questionably decreased autofluorescence (QDAF). DDAF referred to the retinal region in which the level of darkness was at least 90% compared with the optic nerve head; QDAF referred to retinal regions displaying 50 to 90% levels of darkness^10^. The level of blackness was subjectively estimated by the two graders, considering previous assessments reporting high reliability of manual DDAF and QDAF identification^11^.

ORT was defined as a round, ovoid, or tubular hyporeflective lesion with a hyperreflective border located in the outer retina, as detected on structural OCT^12^.

We also evaluated the effect of ORT onset on the expansion of the outer retinal atrophy, as detected on structural OCT. In particular, we considered high-resolution structural OCT lines centered on the ORT, and we measured the linear expansion of the outer retinal atrophy, both on the side of the ORT and on the opposite side, comparing the time of onset of the ORT and the last follow-up examination.

Both structural OCT and FAF measurements were performed at the baseline and at the 3-year follow-up examinations. We calculated the number of eyes displaying ORT on structural OCT, as well as assessing the presence of foveal sparing. This was intended as the presence of partially or fully preserved foveal autofluorescence signal, documented by FAF, together with the presence of partially or fully preserved foveal outer retinal bands, documented on structural OCT.

We adopted the follow-up tool provided by the Heidelberg high resolution structural OCT image device to describe the pathogenic mechanism of ORT formation. Emulating the study of Dolz-Marco and colleagues^7^, we assessed the behavior of the external limiting membrane (ELM) at the point where the ORT formed, also taking into account the flat, curved, reflected and scrolled shapes. To assess the timeline and the order of the events leading to the formation of the ORT, we examined separately at baseline the borders with no signs of incipient ORT and the borders where ORT-related modifications were already detectable. Furthermore, we described a lesion simulating ORT on structural OCT, which we named “pseudo-ORT”, characterized by a mixed reflectivity core surrounded by a hyperreflective border without hypertransmission effect, and we assessed its clinical role.

The primary outcome of the study was the description of ORT development and the assessment of its clinical utility in advanced AMD. The secondary aim was the analysis of the correlation with qualitative and quantitative clinical and OCT features, including BCVA value, thickness of RNFL, GCL, IPL, INL, OPL, ONL, RPE, choroid, areas of DDAF and QDAF, and presence of foveal sparing.

The analyses were conducted by the two expert graders (AA, EA) at least twice to calculate reproducibility and repeatability of the measurements. Inter-graders correlation coefficient (ICC) was calculated to evaluate the agreement between the two operators (overall ICC 0.93; range 0.88–0.96; *p* < 0.01).

For the purposes of statistical analysis, age, gender, disease duration, MNV complication and concomitant diseases (for example hypertension and diabetes mellitus) were considered as fixed factors. In our linear mixed models, we evaluated the normality distribution of each variable with frequency histograms and quantile–quantile plots. Descriptive statistics of continuous variables were reported as mean ± standard deviation, whereas frequency and proportions were described as categorical variables. For each patient included in the study we considered only one eye, randomly selected to offset the possibility of bilaterality. The analysis also incorporated data concerning the atrophy’s progression, obtained by gauging the difference between 3-year and baseline areas of DDAF and QDAF (delta-DDAF and delta-QDAF, respectively). Continuous variables were analyzed by means of a two-tailed T test. Further analyses of eyes with or without ORT were conducted separately. Tau-Kendall correlation analysis was adopted to assess the statistical relationship among all the considered variables. The Bonferroni approach was employed to address the question of multiple comparisons. The statistical significance threshold was therefore set taking into account 16 quantitative variables, making needed to reach a p value ≤ 0.003 (0.05/16). The entire statistical analysis was performed with an SPSS software package (SPSS, Chicago, Illinois, USA).

## Results

We collected data from 88 patients affected by advanced AMD. Eighteen patients were excluded owing to the presence of a large number of media opacities (8 eyes) or because they failed to take part in the follow-up (10 eyes). Thus, 70 eyes of 70 patients (mean age 78 ± 9 years; 31 males) affected by advanced AMD were included in the study. Forty-three eyes were characterized by geographic atrophy, while 27 eyes were affected by MNV. More specifically, 14 MNV were type 1 (52%), 8 mixed type (30%), and 5 type 2 (17%).

Overall, baseline BCVA was 0.5 ± 0.5 LogMAR, deteriorating to 0.9 ± 0.5 LogMAR after 3 years. Foveal sparing was present in 36/70 eyes at baseline (51%) and in 21/70 eyes (30%) at the end of the follow-up (*p* < 0.01). DDAF area was 3.1 ± 2.1 mm^2^ at baseline, increasing to 8.3 ± 4.4 mm^2^ after 3 years (*p* < 0.01). QDAF area started from 11.5 ± 5.2 mm^2^ at baseline, decreasing to 9.6 ± 5.5 mm^2^ at the end of the follow-up (*p* < 0.01). Complete clinical and autofluorescence data are fully reported in Tables 1 and 2 gives all the structural OCT data. We registered a statistically significant reduction in the thickness of GCL (38 ± 12 µm to 33 ± 13 µm), RPE (57 ± 15 µm to 47 ± 14 µm) and SLT (45 ± 17 µm to 38 ± 16 µm) at the end of the follow-up (*p* < 0.01).

**Table 1:** Clinical and fundus autofluorescence data.

	AMD eyes	No ORT	ORT	*p* Value
**Clinical and fundus autofluorescence data**				
No of eyes	70	44	26	> 0.05
Age	78 ± 9	80 ± 8	76 ± 8	> 0.05
Gender M/F	31/39	17/27	14/12	> 0.05
Wet/Dry AMD	27/43	23/21	20/6	< 0.01*
No of IV	6 ± 8	6 ± 9	6 ± 5	> 0.05
LogMAR BCVA 0	0.5 ± 0.5	0.6 ± 0.5	0.5 ± 0.4	> 0.05
LogMAR BCVA 1y	0.7 ± 0.5	0.7 ± 0.5	0.7 ± 0.4	> 0.05
LogMAR BCVA 2y	0.8 ± 0.5	0.8 ± 0.5	0.9 ± 0.5	> 0.05
LogMAR BCVA 3y	0.9 ± 0.5	0.8 ± 0.5	0.9 ± 0.4	> 0.05
*p* value 0 vs 3y	< 0.01*	< 0.01*	< 0.01*	
Foveal sparing 0	36 (51%)	22 (50%)	14 (54%)	> 0.05
Foveal sparing 3y	21 (30%)	16 (36%)	5 (19%)	< 0.01*
p value 0 vs 3y	< 0.01*	< 0.01*	< 0.01*	
Pseudo-ORT	16 (23%)	8 (18%)	8 (31%)	> 0.05
DDAF area 0	3.1 ± 2.1	3.5 ± 2.2	2.4 ± 1.6	> 0.05
DDAF area 1y	4.5 ± 2.5	4.6 ± 2.7	4.2 ± 2.2	> 0.05
DDAF area 2y	6.3 ± 3.4	6.3 ± 3.4	6.4 ± 3.3	> 0.05
DDAF area 3y	8.3 ± 4.4	8.1 ± 4.2	8.6 ± 4.7	> 0.05
*p* value 0 vs 3y	< 0.01*	< 0.01*	< 0.01*	
Delta DDAF %	277 ± 309	204 ± 220	401 ± 394	< 0.01
QDAF area 0	11.5 ± 5.2	11.7 ± 4.9	11.1 ± 5.6	> 0.05
QDAF area 1y	11.7 ± 5.5	13.1 ± 5.8	9.4 ± 4.1	< 0.01*
QDAF area 2y	10.9 ± 5.1	12.0 ± 5.4	9.1 ± 3.7	< 0.01*
QDAF area 3y	9.6 ± 5.5	10.8 ± 6.1	7.4 ± 3.6	< 0.01*
*p* value 0 vs 3y	< 0.01*	< 0.01*	< 0.01*	
Delta QDAF %	− 12 ± 45	− 4 ± 46	− 25 ± 42	> 0.05

Statistically significant *p* values are further highlighted by asterisks.

The following abbreviations are used: intravitreal injections (IV), outer retinal tubulations (ORT), age-related macular degeneration (AMD), best corrected visual acuity (BCVA), definitely decreased autofluorescence (DDAF) area, questionably decreased autofluorescence (QDAF) area.

**Table 2:** Structural OCT data.

	AMD eyes	No ORT	ORT	*p* Value
**Structural OCT data**				
CMT 0	284 ± 67	277 ± 71	296 ± 57	> 0.05
CMT 3y	285 ± 93	289 ± 114	278 ± 41	> 0.05
*p* value	> 0.05	> 0.05	> 0.05	
RNFL 0	33 ± 14	31 ± 10	36 ± 19	> 0.05
RNFL 3y	32 ± 13	32 ± 12	31 ± 14	> 0.05
*p* value	> 0.05	> 0.05	> 0.05	
GCL 0	38 ± 12	37 ± 10	41 ± 14	> 0.05
GCL 3y	33 ± 13	30 ± 10	38 ± 15	< 0.01*
*p* value	< 0.01*	< 0.01*	> 0.05	
IPL 0	41 ± 11	41 ± 9	40 ± 13	> 0.05
IPL 3y	40 ± 38	38 ± 10	44 ± 12	< 0.01*
*p* value	> 0.05	> 0.05	> 0.05	
INL 0	34 ± 9	34 ± 9	33 ± 9	> 0.05
INL 3y	33 ± 9	31 ± 9	35 ± 7	> 0.05
*p* value	> 0.05	> 0.05	> 0.05	
OPL 0	31 ± 12	33 ± 13	26 ± 8	> 0.05
OPL 3y	29 ± 11	27 ± 10	32 ± 12	< 0.01*
*p* value	> 0.05	< 0.01*	> 0.05	
ONL 0	47 ± 15	45 ± 16	50 ± 14	< 0.01*
ONL 3y	41 ± 15	41 ± 16	42 ± 13	> 0.05
*p* value	> 0.05	> 0.05	> 0.05	
RPE 0	57 ± 15	55 ± 12	60 ± 18	> 0.05
RPE 3y	47 ± 14	47 ± 14	48 ± 14	> 0.05
*p* value	< 0.01*	< 0.01*	< 0.01*	
CT 0	172 ± 66	178 ± 65	163 ± 68	> 0.05
CT 3y	162 ± 65	162 ± 70	161 ± 56	> 0.05
*p* value	> 0.05	> 0.05	> 0.05	
HLT 0	127 ± 57	130 ± 56	123 ± 59	> 0.05
HLT 3y	123 ± 57	123 ± 59	124 ± 55	> 0.05
*p* value	> 0.05	> 0.05	> 0.05	
SLT 0	45 ± 17	48 ± 18	41 ± 14	> 0.05
SLT 3y	38 ± 16	39 ± 18	37 ± 12	> 0.05
*p* value	< 0.01*	< 0.01*	> 0.05	

The following abbreviations are used: outer retinal tubulations (ORT), age-related macular degeneration (AMD), central macular thickness (CMT), retinal nerve fiber layer (RNFL) thickness, ganglion cell layer (GCL) thickness, inner plexiform layer (IPL) thickness, inner nuclear layer (INL) thickness, outer plexiform layer (OPL) thickness, outer nuclear layer (ONL) thickness, retinal pigment epithelium (RPE) thickness, choroidal thickness (CT), Sattler layer thickness (SLT), Haller layer thickness (HLT).

We detected completely formed ORT in 26/70 eyes (37%) at the end of the follow-up, with a further 7 eyes (10%) revealing incomplete ORT. The presence of MNV was significantly associated with a greater tendency to develop ORT. Indeed, 20 out of 27 MNV eyes (74%) developed ORT, whereas only 6 out of 43 GA eyes developed ORT (14%) (*p* < 0.01). It took a mean of 18 ± 8 months (range 3–35 months) for ORT to form completely. Fifteen eyes (58%) at baseline disclosed variable ELM alterations (flat ELM in 1 eye, 7%; curved ELM in 2 eyes, 13%; reflected ELM in 5 eyes, 33%; and scrolled ELM in 7 eyes, 47%), whereas 11 eyes (42%) showed no alterations at the atrophic border. However, some ELM alterations were also detectable in the 44 eyes not developing ORT, (flat ELM in 26 eyes, 59%; curved ELM in 13 eyes, 30%; reflected ELM in 3 eyes, 7%; and scrolled ELM in 2 eyes, 4%).

The mean time required to complete ORT formation varied significantly among these eyes, ranging from 5.8 months in scrolled ELM eyes and 12.5 months in reflected ELM eyes to 20.8 months in curved ELM eyes and 30 months in flat ELM eyes.

**Figure 1:**
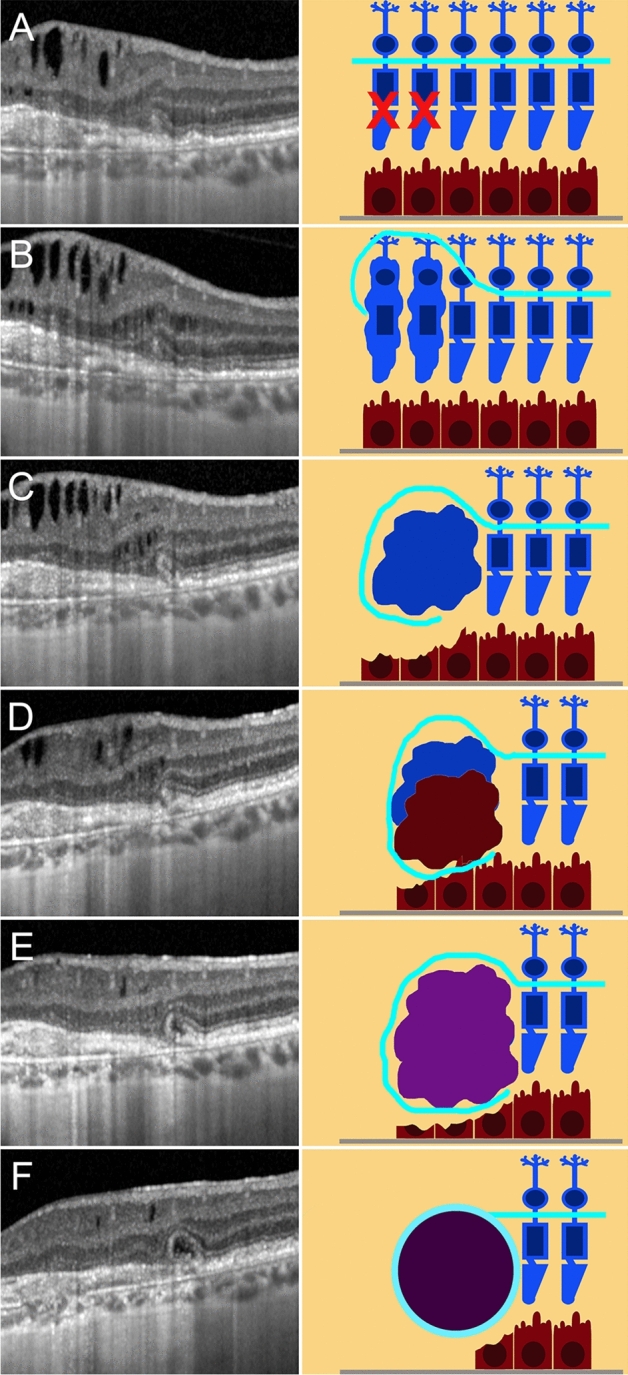
Simplified pathogenic mechanism of ORT development. The first step is primary photoreceptor damage (**A**), with the ELM assuming a curved shape in the proximity of photoreceptor alteration. As the photoreceptors degenerate, ELM assumes an even more evident reflected shape (**B**). ELM increases in reflectivity and thickness around the degenerated photoreceptors, becoming even more organized and assuming a scrolled shape (**C**). At the same time, the RPE cells undergo initial damage, already highlighted by an increased window effect detected at the choroidal level (**C**). RPE and remnants of the photoreceptors become partially fused in the context of the reflected ELM shape (**D**). It is worth noting that pigment is still present, as shown by the lack of window effect below the partially formed ORT (**D**). The next step is characterized by a centrifugal retraction of the partially developed ORT, with complete loss of the pigment and increased outer retinal atrophy extending below the ORT (**E**). The last step is characterized by a completely developed ORT in the ONL, well separated by a hyperreflective border, which is linked to the preserved ELM and characterized by expanded outer retinal atrophy, with hypertransmission reaching up to below the ORT (**F**).

**Figure 2:**
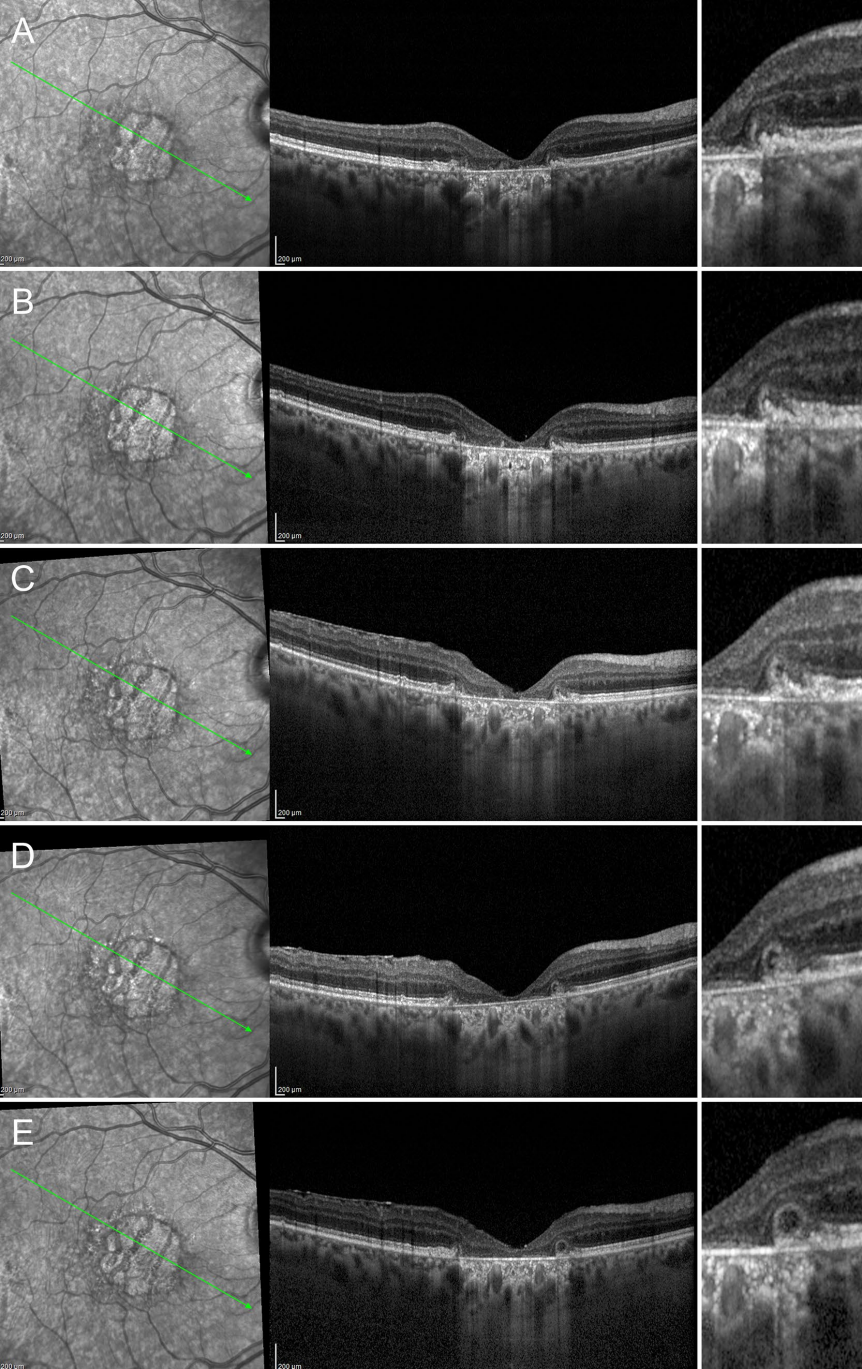
ORT development in AMD complicated by geographic atrophy, showing a curved ELM in the context of partially preserved RPE and already damaged photoreceptor layer (**A**). ELM assumes a reflected shape with initial centrifugal contraction, leading to a first progression of the atrophy (**B**). This contraction– well detected considering the hypertransmission effect—becomes even more evident with a partial organization of RPE, degenerated photoreceptor material and further expansion of the atrophy (**C**). In (**D**) the ORT is partially developed, with completely degenerated RPE, characterized by a complete loss of pigment, leading the window effect to extend below the ORT. In (**E**) we see completely developed ORT in association with outer retinal atrophy.

**Figure 3:**
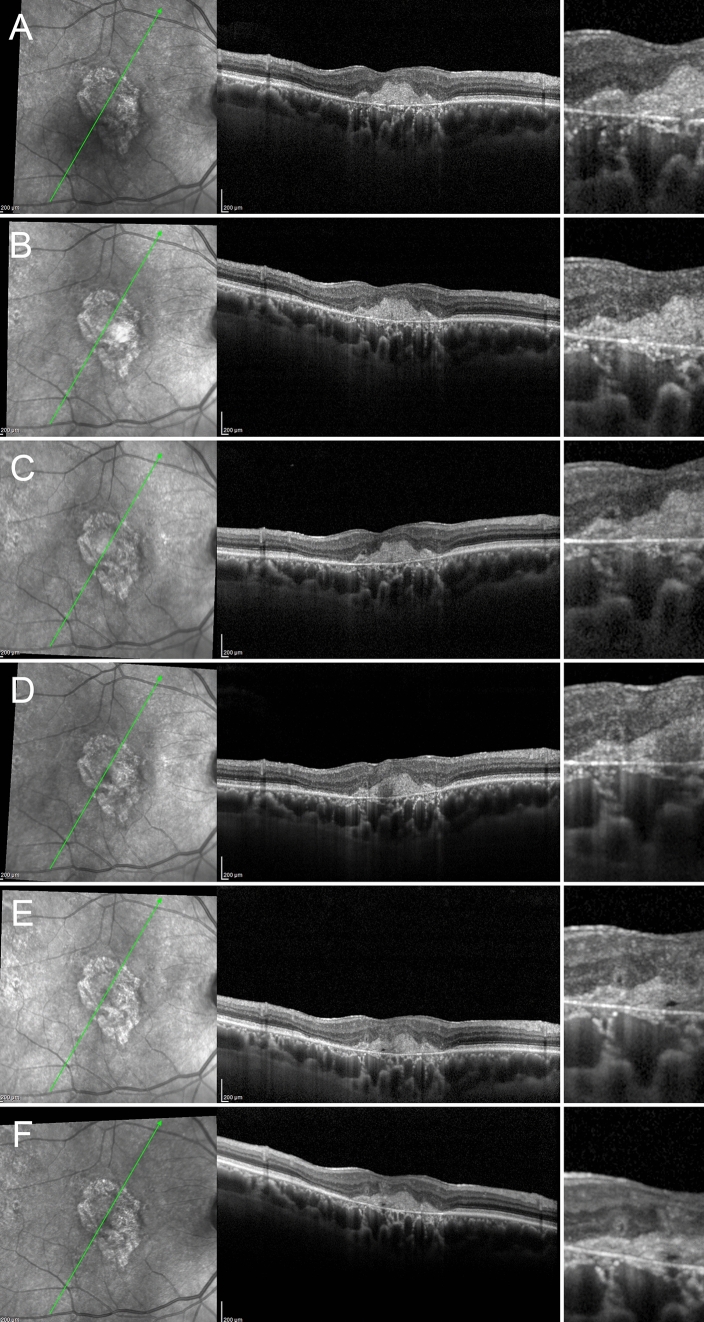
ORT development in AMD complicated by MNV. The initial condition is characterized by a hyperreflective subretinal lesion without exudation, displaying a partially preserved ELM and fused outer retinal bands (**A**). ELM assumes a curved shape (**B**), increasing in thickness and reflectivity (**C**). The partially developed ORT is characterized by the reflected shape of the ELM and the onset of a hypertransmission effect below the lesion (**D**). This light scattering increases in the next follow-up, as the pigment loss proceeds (**E**). Lastly, the image shows a completely developed ORT associated with an inactive MNV lesion with complete fibro-atrophic evolution.

We carefully followed up eyes with no signs of ORT at baseline, noting how the various steps in ORT formation were precisely delineated, as shown by structural OCT images. The first step was the initial degeneration of the photoreceptors bands in the context of preserved ELM, which may appear flat or already curved, with preserved RPE. The ELM revealed a dynamic process of rearrangement, surrounding the gradually degenerating photoreceptors, becoming increasingly curved, later reflected, and finally scrolled. The next step involved the RPE, with RPE migration and atrophy occurring at the same time. The migrating RPE cells progressively merged with remnants of the photoreceptors, with a still evident contribution of the ELM in creating a hyperreflective contour. At this point, the partially formed ORT took on an even more clearly defined circular appearance, while the atrophic border, well-delineated by the hypertransmission effect shown by structural OCT, had notably progressed. A diagram showing the pathogenic progression of ORT development is provided in Fig. 1. The sequence of events leading to the development of ORT described above was similar in both dry and wet AMD (Figs. 2 and 3, respectively).

**Figure 4:**
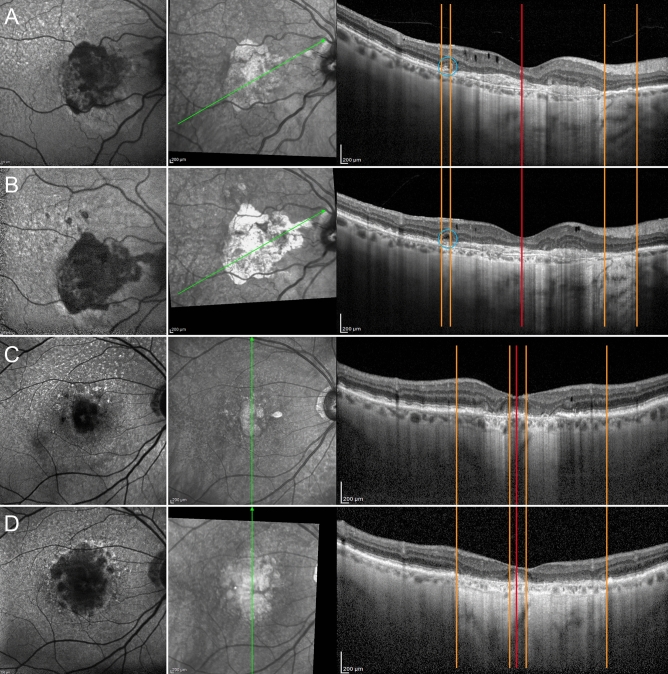
The effect of ORT on the linear expansion of the outer retinal atrophy. In the first case, the onset of ORT (blue circle) (**A**) is associated with an asymmetric linear expansion of the outer retinal atrophy, included between orange lines, revealing itself to be considerably smaller on the side of the ORT, compared with the contralateral side (**B**). In contrast, a case with no onset of ORT shows an almost symmetric progression of the outer retinal atrophy over the follow-up (**C** and **D**, respectively).

Stratified analysis of eyes with or without ORT revealed that the QDAF area was significantly larger in eyes without ORT (*p* < 0.01). On the other hand, DDAF was similar in both subgroups (*p* > 0.05) (Table 1). Eyes presenting with ORT were characterized by less foveal sparing over the follow-up (Table 1) (*p* < 0.01). Structural OCT quantitative analysis found that the following layers in eyes without ORT had worse final thickness than eyes with ORT: GCL, IPL and OPL (*p* < 0.01). Furthermore, eyes without ORT displayed a thinner ONL at baseline (*p* < 0.01), proving to be similar to eyes with ORT at the 3-year follow-up (*p* > 0.05). The correlation analysis confirmed the relationship between the presence of ORT and a stable QDAF area over the follow-up (Tau-Kendall coeff. − 0.243; *p* < 0.01), whereas no significant relationship was found with regard to DDAF changes (*p* > 0.05). This finding was confirmed by measuring the linear expansion of the outer retinal atrophy. Indeed, starting from the completely formed ORT, we observed a statistically significant lower linear extension of the atrophy on the side of the ORT, compared with the opposite side of the structural OCT line (226 ± 189 µm vs 401 ± 237 µm; *p* < 0.01) (Fig. 4).

**Figure 5:**
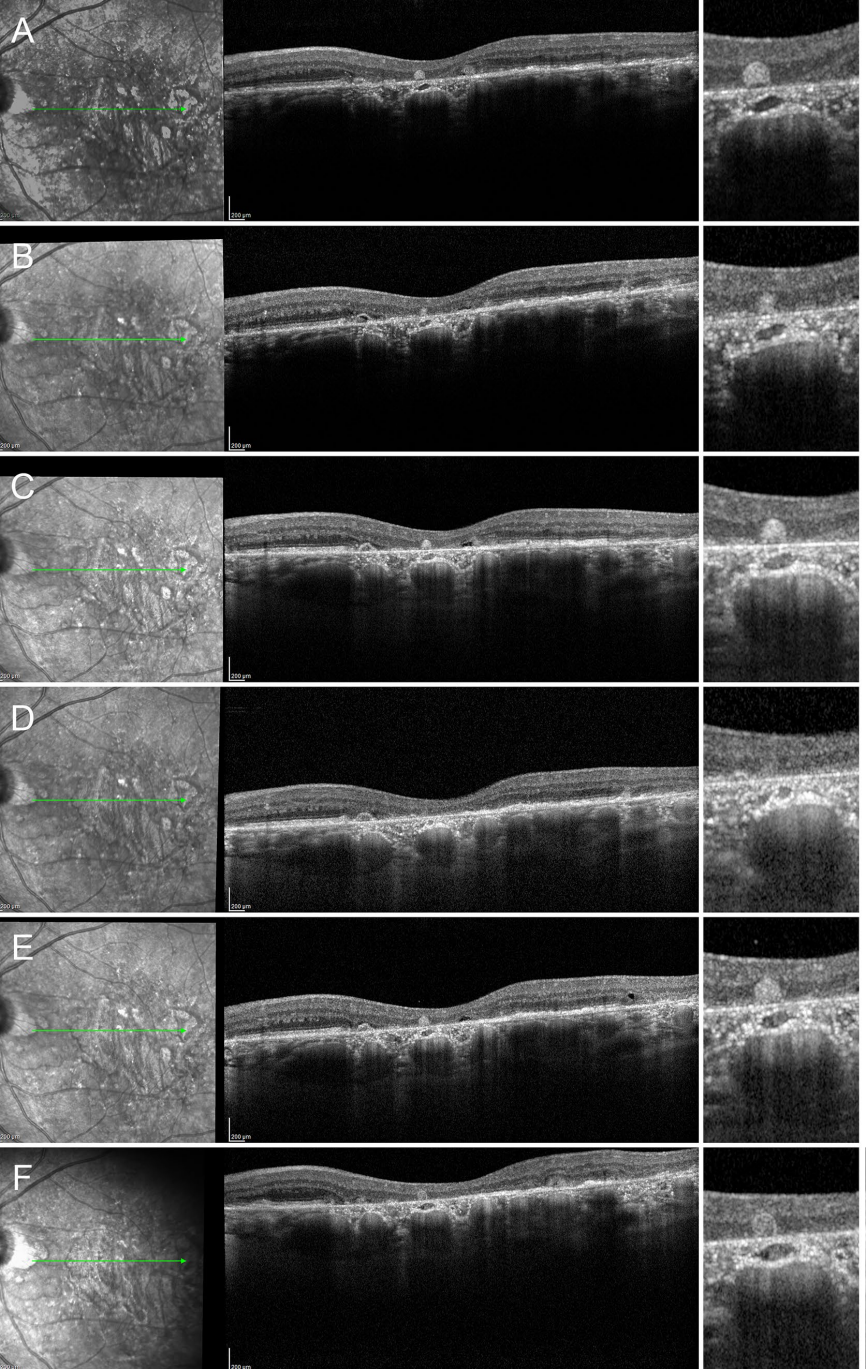
Pseudo-ORT development in AMD, displaying, at baseline, a multiple focal pigment hypertrophy with pigment accumulations in association with complete outer retinal atrophy (**A**). It is worth noting the complete absence of photoreceptor band and ELM. The dynamic rearrangements of this accumulation can be described over time, passing from partial reabsorption (**B**) to fresh increase (**C**), almost complete reabsorption (**D**) and reappearance of accumulation (**E**). Interestingly, no evident hypertransmission window effect can be detected over the entire follow-up, thus reinforcing the interpretation of the lesion as pigment accumulation. In the last image, the reflectivity of the lesion’s content decreases, while the hyperreflective border remains, simulating ORT, although still in the absence of a hypertransmission effect (**F**).

Pseudo-ORT, identified in 16 eyes (23%), showed no ELM or photoreceptor involvement, proving to have been formed only by the RPE, with dynamic hypertrophy and pigment reabsorption processes, as shown in Fig. 5. As in ORT, the presence of pseudo-ORT was associated with less foveal sparing (Tau-Kendall coeff. − 0.356; *p* < 0.01) and a smaller QDAF area at the 3-year follow-up (Tau-Kendall coeff. − 0.206; *p* < 0.01), together with a larger DDAF area (Tau-Kendall coeff. − 0.288; *p* < 0.01).

ORT, pseudo-ORT, DDAF and QDAF measurements showed no significant effects related to age or disease duration (*p* > 0.05). Likewise, the presence of ORT and pseudo-ORT was found to exert no cumulative effect on the fundus autofluorescence or structural OCT quantitative measurements (all *p* > 0.05). Looking at ELM features, the presence of flat, curved, reflected and scrolled ELM shapes appeared to have no significant effect on the progression of the disease (*p* > 0.05). No statistically significant relationship was found between choroidal status and ORT development.

## Discussion

The presence of ORT has been recorded in relation to several retinal diseases^1^, and has been associated with the reorganization of RPE, photoreceptor and Müller cells^7^. Although ORT is already considered a clinically useful biomarker, its role in prognosis is not yet fully established. In this study, we assessed the relationship between ORT and advanced AMD outcomes.

ORT was detected in 37% of our AMD patients over a follow-up period of three years. ORT proved to be significantly more frequent in wet AMD than in geographic atrophy (77% vs 23% of cases), with no significant effects related to age or disease duration. The prevalence of ORT in the literature ranges from 17.4% in the CATT study, covering 2-years of follow-up^12^, and 22% in the 2015 investigation by Hariri et al., in GA, to 41.6% in a 4-year prospective study by Dirani et al., also in 2015^13,14^.

The clinical interpretation of ORT is controversial. The CATT study associated the presence of ORT with poor visual acuity in eyes with neovascular AMD and faster atrophy expansion in eyes with GA^12,13^. On the contrary, other investigations associated ORT with slower atrophy growth^14^. The review performed by Fleckenstein and colleagues summarized the conflicting results as well as the histologic descriptions, reporting no strong relationship between the presence of ORT and atrophy progression^15^, thus leaving open the debate regarding the clinical meaning of ORT in the evolution of advanced AMD.

Based on our findings, the presence of ORT was associated with a smaller QDAF area and similar DDAF area, compared with eyes without ORT. Interestingly, although the foveal sparing rate was similar in eyes with and without ORT at baseline, it was found to be significantly worse in eyes with ORT, with no variation in mean BCVA, after three years of follow-up. Furthermore, on structural OCT, eyes with ORT revealed better GCL, IPL, OPL thickness values after three years.

The stability of the QDAF area, together with the presence of better GCL, IPL and OPL thickness in eyes with ORT, suggest a slower GA progression rate. This consideration found a support on a recent study showing a better intraretinal vascular status in GA eyes developing ORT, compared with GA without ORT, as assessed by optical coherence tomography angiography^8^. In addition, the reduced rate of foveal sparing in eyes with ORT matches the CATT conclusions, which identified an association between ORT and worse visual acuity^12^. The difference in the atrophy’s progression at the periphery and the center (with foveal sparing), suggests two separate pathogenic mechanisms are at work.

Our data complement the observations of Dolz-Marco et al. regarding ORT development^7^, particularly concerning the essential presence of ELM, photoreceptors and RPE in ORT formation. Our structural OCT images suggest that the first step in ORT formation is damage to the photoreceptors, with a dynamic contribution of the ELM, which progressively surrounds the residual photoreceptors, giving rise to the hyperreflective ORT wall. RPE cell damage occurs at the same time; the remaining cells migrating into the partially formed ORT, helping to make up its contents. Lastly, we observed a centrifugal contraction of the ELM, with respect to the fovea, corresponding to a centrifugal migration of the definitely developed ORT, with consequent expansion of the outer retinal atrophy. This mechanism seems to be used both in MNV and pure GA AMD eyes. With respect to MNV eyes, we found no remarkable differences in terms of ORT formation in type 1, mixed type and type 2 lesions. Although the number of eyes was relatively small to draw definite conclusions, we might advance the hypothesis that the localization of the MNV (above or below the RPE) does not influence the process of ORT development. Furthermore, since ORT appear in older lesions, already evolved towards fibrosis and atrophy, and considering ORT development mainly occurring at the border of the lesion, we might assume that RPE seemed to actively participate to ORT formation in all MNV types. Fibrotic phenomena, leading to contraction processes of outer retinal structures, seem to have a role in ORT formation, as already postulated by Janse van Rensburg and colleagues^9^. Differently from these authors describing ORT development as highly associated with type 2 MNV, we reported ORT onset in type 1, type 2 and mixed type MNV lesions. Furthermore, most of our ORT cases were characterized by pure GA. These findings lead to the consideration that ORT pathogenesis might be characterized by extremely heterogeneous processes, whose understanding will benefit from further investigations.

Previous histologic studies have already shown that ORT are composed of the residual photoreceptors, RPE and Müller cells^2,16,17^. On the strength of our findings and a previous description provided by Dolz-Marco et al.^7^ Müller cells may be considered key elements in the formation of ORT. However, in contrast to the conclusion drawn by Dolz-Marco and colleagues^7^, we believe the different ELM shapes may represent consecutive stages of a common mechanism in ORT formation.

Interestingly, our quantitative analysis revealed no statistically significant effect of the choroidal status, considering the overall CT, and HLT and SLT separately, suggesting that ORT development is mainly related to intraretinal modifications.

ORT have been interpreted as the consequence of Müller cell gliosis^7^. In this scenario, considering that foveal, perifoveal and extrafoveal retinal regions are characterized by different populations of Müller cells, associated with completely different functions^18^, the relationship between ORT formation, smaller QDAF area and lower foveal sparing rate might be interpreted as the expression of the varying involvement of each Müller cell subtype.

Pseudo-ORT is a novel OCT lesion similar to ORT and is characterized by a mixed reflectivity core surrounded by a hyperreflective border, but with no hypertransmission effect, simulating a partially formed ORT. However, the absence of ELM and photoreceptors in the context of pseudo-ORT, and the highly dynamic turnover, characterized by alternating accumulations of material and reabsorption, allowed us to clearly distinguish this lesion from ORT. Interestingly, pseudo-ORT was found to be significantly related to less foveal sparing and a larger DDAF area at the 3-year follow-up, thus being potentially interpreted as the sign of a more severe involvement of the outer retinal structures.

We are aware that this study labors under several limitations, particularly with regard to the relatively small number of eyes. Moreover, in spite of the prospective design of the study and the strict follow-up, our analyses are exclusively based on static autofluorescence and structural OCT images, from which we tried to piece together the dynamic process characterizing the development of ORT. We are also aware about the recommendations provided by the Classification of Atrophy (CAM) Consensus Meeting^19–22^; however, we consciously decided to not apply this classification to make the study clearer for the readers and easier to be reproduced in other studies. In addition, we are aware that nowadays OCT angiography is an extremely useful tool, which is why future studies should add microvascular information to assess the possible contribution of choriocapillaris impairment in the pathogenesis of ORT formation and foveal sparing. Also, we must admit that our findings would benefit from further, larger prospective studies, including histologic validation, to provide a more thorough understanding of the contribution of the Müller cells in ORT pathogenesis and of the composition of pseudo-ORT. Lastly, although ORT occur in many retinal diseases, we restricted our analysis to AMD, thus making it necessary to perform further studies focused on the role of ORT in other retinal disorders.

## Conclusions

In essence, our data indicate that ELM is constantly involved in ORT formation, suggesting that ORT is a biomarker of slower GA expansion. Pseudo-ORT mimicking ORT may represent an interesting indicator of outer retina atrophy expansion in a subset of patients. Further studies are warranted to analyze ORT as a biomarker of AMD progression.
